# Microwave-Derived Hierarchic Liquefaction of Pentose and Intensified Separation of Furfural

**DOI:** 10.34133/research.1008

**Published:** 2025-11-20

**Authors:** Ruixuan Yao, Xiao Jiang, Jianchun Jiang, Kui Wang

**Affiliations:** ^1^ Institute of Chemical Industry of Forest Products, Chinese Academy of Forestry, Key Laboratory of Biomass Energy and Material, Jiangsu Province, International Innovation Center for Forest Chemicals and Materials, Jiangsu Co-Innovation Center of Efficient Processing and Utilization of Forest Resources, Nanjing 210042, China.; ^2^ Institute of Biomass Science and Engineering, Henan University of Technology, Zhengzhou 450000, China.

## Abstract

A microwave-coupled biphasic solvent system was developed for steerable liquefaction of pentoses and hierarchic separation of furfural by leveraging the dielectric properties of γ-valerolactone (GVL)/NaCl aqueous solution under microwave irradiation, during which more than 85.38 mol% of furfural yield was obtained from xylan at 140 °C for 20 min, compared to that of 78.1 mol% at 150 °C for 120 min under conventional heating conditions. Strikingly, the separation efficiency was improved under microwave conditions with the partition coefficients (*R*) of furfural ranging from 31.1 to 35.68, compared with 22.71 to 29.33 under conventional heating conditions. Moreover, the coupled effect on the substrate from the microwave was also explored. Notably, high heating power during the initial hydrolysis phase enhances the depolymerization of xylan glycosidic bonds, resulting in the rapid release of 87.89 mol% xylose from xylan. Meanwhile, the application of low heating power during the dehydration process effectively facilitates the production of furfural, which is promptly extracted into the organic phase, thereby minimizing product loss. Besides, the feasibility of the process was assessed using real biomass as the substrate, demonstrating low energy consumption attributed to the efficient microwave absorption by GVL and the NaCl aqueous solution. This approach offers a viable strategy for the efficient and scalable production of furfural.

## Introduction

The environmental issues resulting from the extensive fossil fuels use have become increasingly prominent [1]. This underscores the urgent need to develop the circular economy and shift to renewable energy sources. The efficient biomass-to-chemicals conversion represents a crucial foundation of green energy utilization, garnering substantial attention from both academia and industry [2]. Furfural, as a biomass-derived intermediate from pentose [3,4], has been identified by the US Department of Energy as a key platform chemical. Furfural can be hydrogenated to furfuryl alcohol, 2-methylfuran, and 2-methyltetrahydrofuran, valuable solvents and intermediates, or oxidized to furan-2-carboxylic acid for further conversion into ketones and polyols [5–7]. The thermochemical conversion of biomass is closely linked to mass and heat transfer, making solvent optimization, advanced heating techniques, and highly selective catalysts crucial.

Conventional single-phase routes to furfural use water or polar aprotic solvents but suffer inherent drawbacks. Water serves as a green solvent and hydrogen donor in catalytic dehydration, yet it also accelerates furfural degradation and limits overall yield [8]. Organic solvents can improve biomass conversion and furan selectivity [9]. However, single-phase reactions still face severe side reactions, difficult product recovery, and poor catalyst recyclability [10]. In contrast, a biphasic system employs an aqueous reaction medium paired with an immiscible organic extraction solvent to extract furfural as it forms, suppress secondary condensations, and simplify downstream separation. Meanwhile, the strong attraction of Na^+^ to H_2_O molecules compared to organic solvents allows NaCl to facilitate phase separation and enhance the extraction efficiency of the furfural, collectively boosting yield and process efficiency [11]. However, heating methods for producing furfural have primarily focused on conventional heating approaches dominated by heat conduction, such as high-pressure reactors and hydrothermal reactors. This method leads to an uneven temperature distribution and does not fully exploit the synergistic effects of the solvent’s dielectric properties and the heating technique employed. Due to the low thermal conductivity of the solvent, heat transfer efficiency is limited, which indirectly promotes the formation of humins and thus prevents further increases in furfural yield [12]. Therefore, it is necessary to establish a microwave-coupled reaction system to fully leverage the properties of the solvent under microwave conditions, thereby further enhancing the yield of the product.

Microwave-reactant interactions convert electromagnetic energy into heat at the molecular level through the rapid rotation of polar molecules (dielectric polarization) and the rapid migration of ions (ionic conduction) within oscillating electromagnetic fields [13]. This mechanism facilitates microwave–solvent coupling, resulting in uniform heating and expedited reactions. As a consequence, reaction times are shortened, energy consumption is reduced, and product yields are improved [14]. Yang et al. [15] demonstrated that switching to microwave heating in an H₂O/tetrahydrofuran biphasic system with AlCl₃·6H₂O and NaCl raised furfural yields from 38% under conventional heating to 64%. In microwave-coupled biphasic systems, each solvent phase absorbs microwave energy at a specific frequency, with its responsiveness depending on dielectric properties [16,17]. The dielectric constant (*ε*′) measures a material’s capacity to store electromagnetic energy, the dielectric loss (*ε*″) quantifies its ability to convert that energy into heat, and their ratio, tan*δ* (*ε*″/*ε*′), serves as a measure of heating efficiency [18,19]. In recent years, microwave has been widely applied in thermochemical transformations. However, most existing reports merely substitute microwave for conventional heating methods without designing or screening systems tailored to microwave characteristics. For instance, Yang et al. only evaluated the catalytic performance of different catalysts under microwave heating. In a γ-valerolactone (GVL)/NaCl aqueous system using xylan as substrate, the reaction yielded only 72.3% furfural for 50 min, without experimental design considering microwave-specific factors [20]. Currently, microwave-assisted thermochemical conversion of biomass remains at the laboratory scale. In fact, rationally designing reactions based on product characteristics and developing temperature measurement equipment adapted for microwave environments will facilitate the industrialization of microwave-assisted biomass thermochemical conversion processes [21]. Accordingly, to maximize furfural yield, it is essential to develop a highly efficient and stable system that integrates microwave energy with the specific characteristics of the reactants. This microwave-coupled reactant system is designed to optimize the utilization of microwave energy.

In furfural synthesis, both heterogeneous and homogeneous catalysts play a crucial role. For example, a Starbon450-SO_3_H solid acid in cyclopentyl methyl ether (CPME)/H_2_O achieved a 70 mol% yield from xylose [22]. Although heterogeneous catalysts allow easy separation, they often require complex preparation, suffer from poor thermal stability, and find active-site blockage by humins [23]. By contrast, simple homogeneous acids excel in biphasic media: the yield of furfural produced from xylose, catalyzed by H₂SO₄ in both H_2_O/methyl isobutyl ketone (MIBK) and H_2_O/toluene (TL), reached 80.1% and 76.3%, respectively [24]. In addition to protonic acids, transition metal salts also exhibit excellent performance in furfural production. Literature reports indicate that in an H₂O/TL system combined with NaCl, CrCl₃ can effectively convert corn cob into 23.9% furfural [25]. More recently, ionic liquids and deep-eutectic solvents have shown promise: a choline-chloride/formic acid deep eutectic solvent (DES) with SnCl_4_ cocatalyst gave 49.6% furfural, as SnCl_4_ disrupts DES interactions and promotes reactant availability [26–29]. Nevertheless, research on the conversion of pentoses to furfural has primarily concentrated on catalyst and solvent systems. The mechanism by which coupling energy fields with the properties of solvents and feedstocks enhances furfural yield remains underexplored and warrants further investigation.

**Fig. 1.**
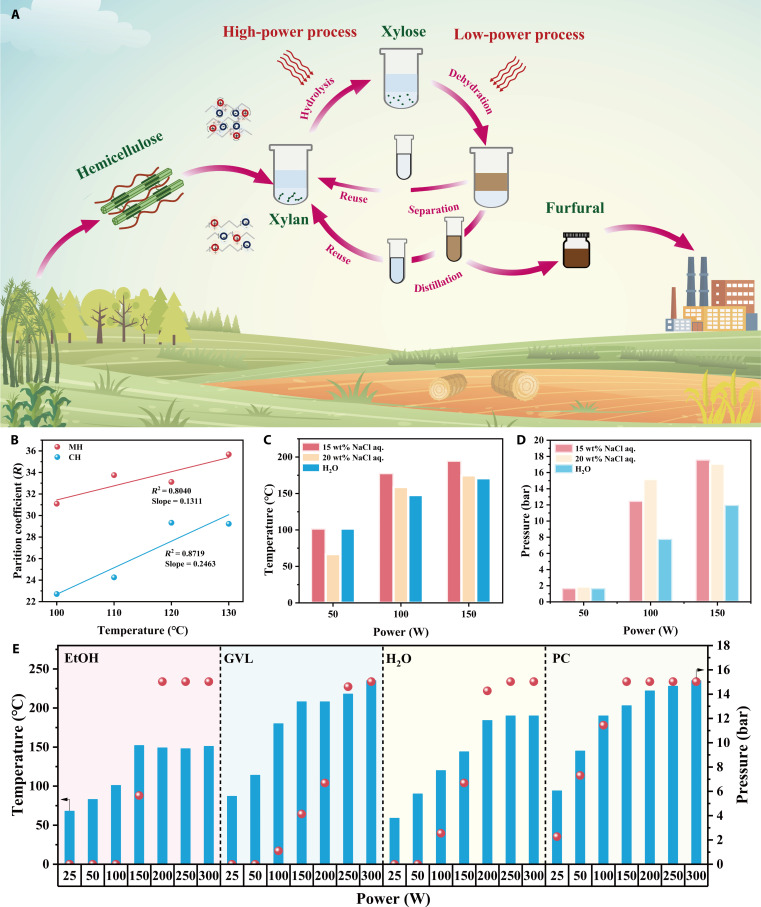
(A) Schematic illustration of high-value utilization of furfural under microwave conditions. (B) Partition coefficients (*R*) of furfural under microwave heating and conventional heating. The power consumed by different concentrations NaCl aq. to reach (C) temperatures and (D) pressures. (E) The power consumed by organic solvents to reach temperatures and pressures.

In this study, we establish a microwave-coupled solvent system for the efficient conversion of xylan into furfural. All experiments were performed using uniform microwave radiation heating, as confirmed by the consistent reaction pressure (Fig. S1). By comparing the differences in partition coefficients of furfural under microwave and conventional heating conditions, the mechanism by which microwave irradiation promotes in situ extraction of furfural was investigated. Seven organic solvents (propylene carbonate [PC], MIBK, ethyl acetate [EA], ethanol [EtOH], GVL, TL, and water) were evaluated alongside aqueous NaCl solutions (5 to 20 wt%) for their microwave absorption characteristics, focusing on microwave responsiveness and solvent vapor pressure. This aimed to identify solvent systems that maximize microwave coupling, yielding certain patterns and conclusions. The GVL/NaCl mixture delivered the optimal balance of rapid heating and product partitioning, making it the solvent system with the highest coupling efficiency when used with microwaves. The effect of microwave power on reaction substrates highlights its role in promoting the depolymerization of xylan glycosidic bonds and facilitating the isomerization–dehydration conversion process. Kinetic studies elucidate the mechanism behind the differential production of furfural from xylose and xylan, emphasizing the interaction between microwave energy and the substrates. Reaction temperature, time, GVL/NaCl ratio, and catalyst loading were systematically optimized to maximize furfural yield and selectivity. Electrospray ionization mass spectrometry confirmed the identity of the active tin species. Solvent and catalyst recyclability tests demonstrated the system’s biorefinery potential (Fig. 1A). The application of biomass conversion under microwave irradiation, along with comparison of the energy consumption between microwave and conventional heating, underscores the promising potential of microwave technology.

## Results and Discussion

### Initial insights into microwave-assisted organic solvent extraction

To assess the differences in partition coefficients of furfural under microwave versus conventional heating, the behavior of furfural under both conditions was investigated. Furthermore, previous research indicates that GVL notably protects furfural and contributes to the stabilization of the active sites of Lewis acids. The NaCl-regulated nonclassical biphasic system provides distinct advantages, including enhanced furfural yield and high tunability [26,30–33]. Therefore, in initial work, we evaluated the partitioning behavior of furfural in a biphasic GVL/NaCl aqueous solution (aq.) system. Due to the differing binding energies between furfural, Na^+^, and GVL in pairwise interactions, furfural preferentially partitions into the GVL phase even without heating [30]. This tendency is further enhanced by heating. A fixed amount of furfural (100 mg) was added to the biphasic mixture consisting of 2 ml of 15 wt% NaCl aq. and 6 ml of GVL. The system was subjected to both microwave irradiation and conventional oil-bath heating at identical temperatures and durations. To quantify the extraction efficiency in different heating modes, we defined the parameter *R* as the ratio of furfural concentration in the organic phase to that in the aqueous phase:

$$R=\frac{C_{\mathrm{org}}}{C_{\mathrm{aq}}}$$ (1)

where *C*_org_ and *C*_aq_ are the concentrations of furfural in the organic and aqueous phases, respectively. As shown in Fig. 1B, within the temperature range of 100 to 130 °C, the *R* value under microwave heating consistently exceeded that obtained with conventional heating. This can be attributed to the direct interaction of microwave energy with polar molecules and ions, which promotes rapid internal energy transfer. Unlike thermal conduction, microwave irradiation allows molecules to quickly absorb the energy from the electromagnetic field, facilitating faster redistribution of furfural between the phases and reaching equilibrium, especially at lower temperatures (e.g., 100 °C). Furthermore, the differing dielectric properties between the 2 phases create a temperature gradient that may also contribute to enhancing the transfer of furfural from water to GVL [34]. These results suggest that microwave irradiation notably enhances the extraction efficiency of furfural in biphasic systems.

In addition to improving furfural extraction efficiency, microwave heating underscores the importance of solvent selection for process safety and sustainability. To identify an optimal solvent system, we systematically evaluated classical biphasic mixtures combining NaCl aq. with various organic solvents, considering microwave absorption, heating performance, and safety. The addition of NaCl introduces an ion conduction mechanism during microwave heating, particularly in dilute solutions, which accelerates heating rates and induces localized superheating. To quantitatively assess this effect, we conducted constant-power microwave heating experiments on NaCl solutions of varying concentrations. We monitored both the maximum temperature achieved (Fig. 1C and Fig. S2A) and the corresponding vapor pressure (Fig. 1D and Fig. S2B) at a fixed power level. At lower NaCl concentrations (5 to 10 wt%), ion conduction heating was particularly pronounced, leading to the formation of micro-hotspots. This effect is evidenced by a notable increase in vapor pressure compared to pure water (at 100 W, pure water: 7.8 bar; 5 wt% NaCl: 17.6 bar). At a NaCl concentration of 15 wt%, the solution attains a temperature of 178 °C when heated with 100 W of power. When the power is increased to 150 W, the temperature of the 15 wt% NaCl solution rises to 195 °C. However, further increases in NaCl concentration attenuated this phenomenon (20 wt% NaCl, at 100 W: 159 °C, at 150 W: 175 °C). At a concentration of 15 wt%, the ionic conduction loss and relaxation loss in the NaCl aq. attain an optimal equilibrium, resulting in the best absorptive properties. At this concentration, *ε*′, *ε*″, and tan*δ* of NaCl aq. are measured at 46.54, 50.26, and 1.08, respectively, at a frequency of 2.45 GHz (Table S1). However, as the concentration of the NaCl aq. increases beyond this point, the absorptive properties decline. This reduction is likely attributed to restricted ion mobility and diminished water dipole polarization at higher ionic strengths [35]. Despite the observed attenuation, NaCl solutions exhibited a higher overall dielectric heating efficiency than pure water, supporting the selection of a 15 wt% NaCl concentration. It not only facilitates the formation of nonclassical biphasic systems but also enables efficient and controllable microwave heating at reduced power levels.

Organic solvents that encompass a range of polarities, including high, medium, and low polarity solvents, have been selected. Among the selected solvents, water, EtOH, and PC are categorized as highly polar solvents; GVL, EA, and MIBK are identified as moderately polar solvents; while TL is classified as a low-polarity solvent. As shown in Fig. 1E and Fig. S2C, both PC and GVL rapidly exceeded 200 °C at low microwave power. In contrast, EtOH and water exhibited moderate heating. EA, TL, and MIBK reached lower maximum temperatures (<130 °C) [36]. The ability of a solvent to absorb microwaves is influenced not only by its polarity but also notably by its tan*δ*. PC and GVL, both classified as polar nonprotonic solvents, exhibit high *ε*′ and very low molecular rotational resistance, resulting in exceptionally high *ε*″. Specifically, *ε*′, *ε*″, and tan*δ* of GVL at 2.45 GHz are 24.71, 13.84, and 0.56, respectively (Table S1). This unique combination allows them to convert microwave energy into heat with greater efficiency [19]. However, low-boiling solvents pose pressure risks under microwave conditions, and PC is further limited by its thermal instability. Among the candidates, GVL required the least microwave power to maintain target temperatures, resulting in high energy efficiency (Fig. S2D and E). This characteristic makes GVL the most promising option for safe and energy-efficient microwave-assisted furfural production.

### Optimization of reaction parameters

**Fig. 2.**
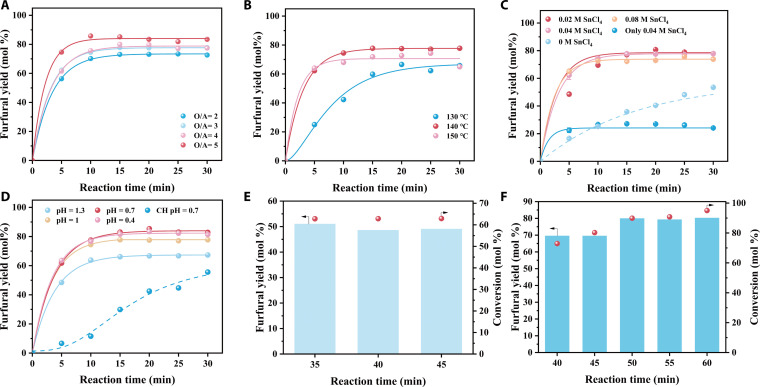
The conversions of xylan to furfural with (A) different solvent ratios, (B) temperature, (C) Lewis acid concentrations, and (D) pH value. The conversions of xylan to furfural (E) without SnCl_4_ and (F) under conventional heating.

Efficient conversion of xylan to furfural in the biphasic GVL/NaCl aqueous system was achieved through systematic optimization of key parameters, including the GVL/NaCl volume ratio, reaction temperature, catalyst loading, pH, and heating method (Fig. 2 and Fig. S3). Among these factors, the GVL/NaCl ratio emerged as a critical determinant. Increasing the GVL volume from 2 to 5 times that of the aqueous phase markedly enhanced furfural yield, achieving a maximum of 85.76 mol% (Fig. 2A). This improvement can be attributed primarily to enhanced extraction efficiency at higher GVL volumes [37]. However, ratios at or above 4:1 (GVL: NaCl aq., v/v) disrupted the biphasic equilibrium, resulting in NaCl precipitation, phase mixing, and complications in catalyst recovery due to mixing with high-boiling solvents. Accordingly, a ratio of 3:1 was identified as optimal, striking a balance between extraction efficiency, phase stability, and economic feasibility.

Temperature also exerted an important influence on process efficiency. An optimal temperature of 140 °C afforded a furfural yield of 77.79 mol% within 15 min with minimal side reactions (Fig. 2B). Operating at 150 °C led to rapid condensation reactions between furfural and xylose, promoting humins formation under microwave conditions, whereas lower temperatures (130 °C) resulted in slower xylan conversion and undesired side reactions. Notably, the reaction system exhibited minimal furfural degradation within the temperature range of 130 to 150 °C and remained stable after achieving peak yield.

In the transformation of furfural, Brønsted acid (HCl) primarily facilitates xylan hydrolysis and xylose dehydration, while the Lewis acid (Sn^4+^) catalyzes xylose-to-xylulose isomerization, the latter being a known rate-determining step. Catalyst screening indicated that a Sn^4+^ concentration of 0.02 M delivered the highest yield (80.7 mol%) through synergistic Brønsted–Lewis acid catalysis (Fig. 2C), surpassing systems utilizing either acid alone. Fine-tuning the reaction phase pH to 0.7 using HCl further improved the furfural yield to 85.38 mol% in 20 min (Fig. 2D). Additionally, microwave irradiation notably enhanced both the reaction rate and selectivity compared to conventional oil-bath heating (Fig. 2E and F and Fig. S4).

### Selection of acidic sites: Metal cation screening

**Fig. 3.**
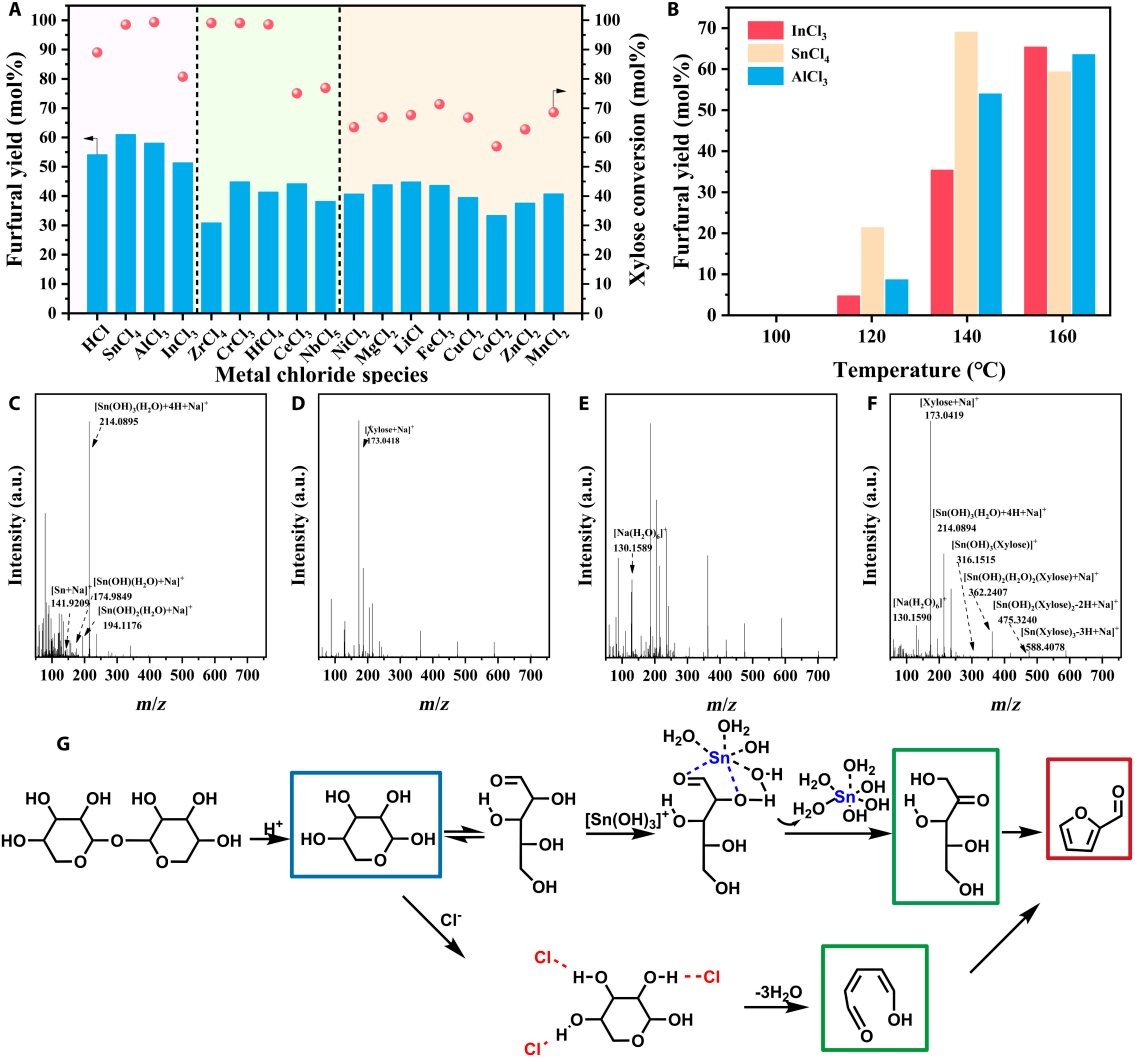
(A) Xylose conversion by using different Lewis acid types. (B) Xylose conversion using 3 catalysts at different temperatures. ESI-MS spectra of (C) the aqueous SnCl_4_ solution, (D) the aqueous xylose solution, (E) the aqueous NaCl solution, and (F) the mixture solution. (G) The proposed reaction mechanism of the conversion of xylan to furfural.

To systematically investigate catalyst performance in the microwave-assisted transformation of xylose to furfural in a biphasic GVL/NaCl aq. system, 16 metal chlorides were examined under uniform conditions (0.2 g of xylose, 0.04 M metal chloride, 2 ml of 15 wt% NaCl aq., 6 ml of GVL, pH = 1 by HCl, 150 °C for 15 min). The results, summarized in Fig. 3A, revealed clear differences tied to the intrinsic properties of the metal ions. Specifically, SnCl_4_, AlCl_3_, and InCl_3_ led to both high xylose conversions (>80 mol%) and high furfural yields (>50 mol%). This superior catalytic activity is attributable to their high Lewis acidity and charge density, which efficiently activate xylose molecules, facilitating both isomerization and subsequent dehydration steps central to furfural production. These strong Lewis acids are thus highly effective even at relatively low temperatures.

In contrast, while HfCl_4_, CrCl_3_, and ZrCl_4_ also promoted high xylose conversion, the corresponding furfural yields were below 45 mol%. Although these metals are also high valence and possess moderate Lewis acidity, their higher propensity for hydrolysis or aggregation in aqueous media can generate less defined catalytic environments. This may enhance undesired side reactions or by-product formation, thereby reducing overall furfural selectivity despite efficient substrate conversion.

On the other hand, NiCl_2_, MgCl_2_, and LiCl resulted in much lower xylose conversions (63 to 67 mol%) and furfural yields. Their relatively weak Lewis acidity and low charge densities confer limited activation of xylose, reducing both conversion and furfural formation. However, these weakly acidic catalysts also suppress side reactions, such as condensation and humin formation. This is evidenced by their higher carbon balances compared to other metal chlorides, indicating a more efficient preservation of reactants within the system.

To further differentiate the high-performing catalysts, reactions with SnCl_4_, AlCl_3_, and InCl_3_ were conducted over a range of temperatures (Fig. 3B and Fig. S5). At 100 °C, only SnCl_4_ yielded notable xylose conversion, demonstrating its superior ability to activate the substrate at lower energy input. Nevertheless, minimal furfural was produced by any of the 3 catalysts at this temperature, underscoring that thermal input is necessary for both sufficient reactant activation and effective dehydration. When the temperature was raised to 120 °C, all 3 catalysts showed increased conversion and furfural yield, with InCl_3_ providing the highest conversion, yet SnCl_4_ yielded the most furfural, indicative of SnCl_4_’s greater selectivity. At 140 °C, SnCl_4_ further excelled, achieving nearly complete xylose conversion (97.93 mol%) and a maximum furfural yield (69 mol%). Notably, at 160 °C, SnCl_4_’s performance declined relative to AlCl_3_ and InCl_3_, likely due to excessive catalysis that promotes the degradation of furfural or the formation of additional by-products. Under rapid microwave heating with immediate quenching at the target temperature, we compared SnCl_4_, AlCl_3_, and InCl_3_ for furfural production from xylan, xylose, and xylulose. As shown in Tables S2 to S4, SnCl_4_ emerged as the most effective catalyst for efficiently hydrolyzing xylan and isomerizing xylose to xylulose and thus delivered the highest furfural yield.

Altogether, these observations demonstrate that the catalytic effectiveness and selectivity of metal chlorides in this system are determined by a balance between Lewis acid strength, hydrolytic stability, and coordination environment. Strong Lewis acids with stable coordination environments, such as SnCl_4_, are optimal for high furfural yields under controlled conditions, while weaker Lewis acids minimize by-products but suffer from low activity [38,39]. Such mechanistic insights are crucial for the rational selection and optimization of catalysts for biomass conversion processes.

### ESI-MS characterization of the aqueous xylose solutions with SnCl_4_, NaCl, and HCl

To further elucidate the catalytic mechanism underlying the conversion of xylan to furfural, it is essential to characterize both the nature and evolution of the active Sn species and to describe the associated reaction pathways. In the aqueous phase of biphasic systems, these Sn species often coexist as multiple hydrolyzed and coordinated forms, whose relative abundances are highly sensitive to pH and other reaction parameters, making their identification challenging. Electrospray ionization mass spectrometry (ESI-MS) was employed to probe Sn speciation under various conditions. As shown in Fig. 3C, the spectrum of SnCl_4_ in acidic medium exhibits peak at *m*/*z* = 141.9, 175.0, 194.1, and 214.1, assignable to [Sn + Na]^+^, [Sn(OH)(H_2_O) + Na]^+^, [Sn(OH)_2_(H_2_O) + Na]^+^, and [Sn(OH)_3_(H_2_O) + 4H + Na]^+^, respectively. Under these conditions, [Sn(OH)_3_]^+^ is the predominant Sn species, with [Sn(OH)_2_]^2+^ present to a lesser extent—consistent with the known hydrolysis equilibria of Sn^4+^ [40].

For comparison, the ESI-MS spectrum of a xylose-only solution (Fig. 3D) shows a single peak at *m*/*z* = 173.0 ([Xylose + Na]^+^), while the neat NaCl solution (Fig. 3E) yields *m*/*z* = 130.2 ([Na(H_2_O)_6_]^+^). In contrast, the mixed solution containing xylose, SnCl_4_, HCl, and NaCl (Fig. 3F) retains both [Xylose + Na]^+^ and [Sn(OH)_3_(H_2_O) + 4H + Na]^+^ signals and exhibits new peaks at *m*/*z* = 316.2, 362.2, 475.3, and 588.4. These latter signals correspond to [Sn(OH)_3_(Xylose)]^+^, [Sn(OH)_2_(H_2_O)_2_(Xylose) + Na]^+^, [Sn(OH)_2_(Xylose)_2_ − 2H + Na]^+^ and [Sn(Xylose)_3_ − 3H + Na]^+^, respectively. Their presence confirms that Sn^4+^ directly coordinates to xylose, forming a series of Sn–xylose complexes that serve as catalytically active intermediates.

Building on these observations and literature precedents, we propose the mechanism depicted in Fig. 3G for xylan conversion in the GVL/NaCl aq. biphasic system. SnCl_4_ hydrolyzes to release H^+^ and produce [Sn(OH)_3_]^+^ catalytic centers. Brønsted acid (H^+^) first depolymerizes xylan to xylose. The [Sn(OH)_3_]^+^ species then coordinates to the C1 and C2 oxygen atoms of xylose, facilitating hydride transfer and accelerating its isomerization to xylulose. Finally, acid-catalyzed dehydration of xylulose yields furfural [41]. Anions also play a crucial role in the reaction mechanism. Cl^−^ interacts with hydroxide ions to facilitate the cleavage of the C1-O6 bond in xylose, enabling ring opening. Additionally, Cl^−^ engages in hydrogen bonding with the hydroxyl groups at positions C2, C3, and C4 (mediated by water and GVL). These interactions foster intermolecular hydrogen bonds that both accelerate dehydration and suppress intramolecular bonding, thereby preventing undesired cross-reactions between substrate hydroxyls and product aldehyde groups [42].

In summary, the synergistic interplay between hydrolyzed Sn-based cations and Cl^−^ in the GVL/NaCl aq. system underpins the highly efficient, selective conversion of xylan to furfural. This mechanistic insight highlights the critical importance of both cation and anion speciation and their cooperative action in dictating overall reaction performance.

### Effects of xylose and xylan substrates on microwave-assisted reaction outcomes

**Fig. 4.**
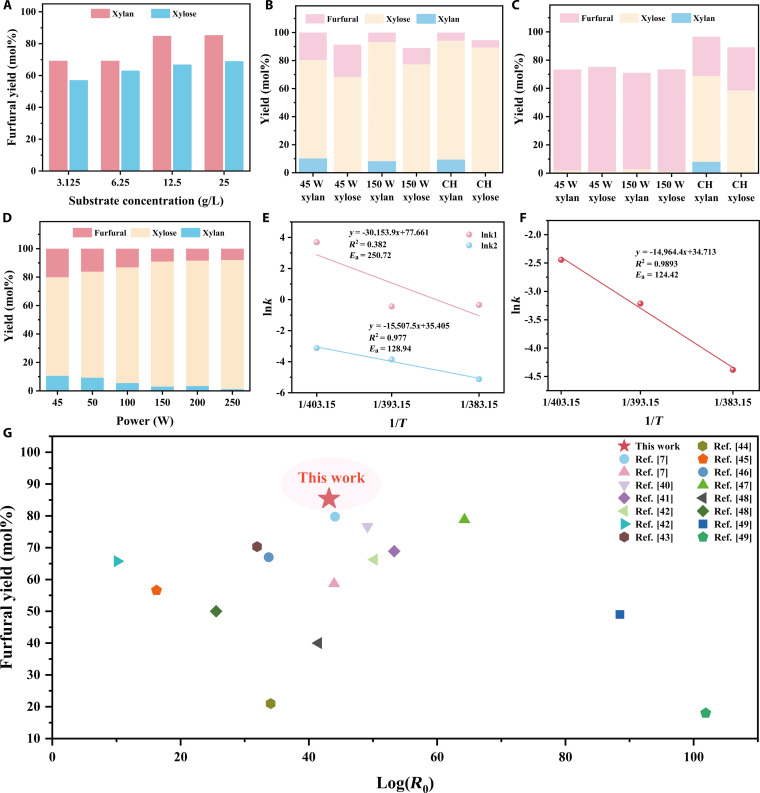
(A) The conversions of xylose and xylan to furfural at different substrate concentrations. The conversions of xylan and xylose to furfural at different powers at a holding time of (B) 0 min and (C) 20 min. (D) The conversions of xylan to furfural at different heating powers. The activation energy (*E*_a_) of (E) xylan and (F) xylose to furfural. (G) Comparison with the results of furfural production process reported in the literature.

In the context of economically viable biomass conversion, direct furfural production from lignocellulosic hemicellulose is far more attractive than using purified xylose. To simulate the behavior of hemicellulose under microwave-assisted conditions, we utilized xylan as the substrate. Our findings revealed that, at equivalent monosaccharide loading (based on xylose released during hydrolysis), xylan consistently produced furfural yields that were 10 to 20 mol% higher than those obtained from pure xylose. This advantage further increased with rising substrate concentrations (Fig. 4A).

To uncover the origin of this discrepancy, we focused on the distinctive power–temperature profile of microwave heating (Fig. 4B and C). Microwaves supply constant power until the target temperature is reached. First, we probed the effect of heating rate by comparing slow heating at 45 W (minimum power to maintain 140 °C, reaching 140 °C in 5.3 min) with rapid heating at 150 W (reaching 140 °C in <1.5 min). Under rapid heating, xylan hydrolysis is greatly accelerated, liberating 85.0 mol% of its xylose during the ramp-up. This amount was substantially higher than the 70.34 mol% of xylose released during slow heating at 45 W. This fast hydrolysis both maximizes xylose availability for subsequent dehydration and limits premature xylose-to-furfural conversion [43–45]. This approach addresses the challenge of low furfural yield associated with condensation reactions. When xylose was utilized as the raw material, the furfural yield was noticeably higher during slow heating at 45 W than during rapid heating at 150 W. This discrepancy can be attributed to the longer duration of the slow heating process, which provides more time for the conversion of xylose into furfural.

Next, we examined the influence of the post-heating hold by maintaining the reaction at 140 °C for 20 min under either 45 or 150 W (using forced cooling to keep the bulk temperature constant). As illustrated in Fig. 4C, the highest furfural yield (73.94 mol%) was achieved from the reaction utilizing xylose as feedstock under low power conditions at 45 W. This was followed by another reaction with xylan at low power (71.41 mol% furfural yield). Conversely, the furfural yield remained consistently low at high power conditions of 150 W (72.4 mol% furfural yield from xylose, 68.21 mol% furfural yield from xylan). This discrepancy is attributed to the fact that low-power maintenance more effectively preserves furfural [46]. To gain a clearer understanding of the impact of heating rate on the catalytic conversion of xylan, the reaction was investigated under heating powers spanning from 45 to 250 W (Fig. 4D). Table S5 presents the heating times at various power levels. It can be observed that under slow heating at 45 W, xylan did not fully release xylose, and furfural had already formed at this stage. As the heating power increased, the amount of xylose hydrolyzed from xylans also rose. However, excessively high power increases the energy consumption of the reaction. In summary, rapid microwave heating accelerates xylan hydrolysis and suppresses early side reactions, while a low-power hold minimizes furfural loss. Optimizing this power–temperature profile is therefore key to efficient furfural production and high energy efficiency under microwave conditions.

We have quantitatively dissected the xylan-to-furfural pathway under microwave irradiation by determining both rate constants (Scheme S1, Tables S6 and S7, and Fig. S6) and their activation energies (*E*_ah_ and *E*_ad_; Fig. 4E and F). Under microwave heating, the activation energy for xylan hydrolysis to xylose (*E*_ah_) is 250.7 kJ mol^−1^, whereas that for xylose dehydration to furfural (*E*_ad_) is only 128.9 kJ mol^−1^ (Fig. 4E). The substantially higher barrier for xylan hydrolysis explains why intense microwave fields are required. Dielectric polarization of the glycosidic bonds lowers the effective barrier, driving rapid chain scission even without a holding period. Consequently, at 150 W, we achieved a xylose release of 87.9 mol% during the ramp-up phase alone. The applied high power facilitates the cleavage of glycosidic bonds in xylan, thereby preventing the condensation of xylose with trace amounts of furfural. As a result, unwanted condensation reactions are effectively suppressed [43,47]. To benchmark our system against the literature, Fig. 4G and Table S8 compare reaction conditions and furfural yield using xylan and xylose. The furfural yield observed in our work is substantially greater than what has been reported in the existing literature. Microwave heating both shortens reaction time and increases yield. log*R*_0_ represents the degree of treatment, with detailed equations provided in Eqs. S3 to S5 [8,48–57].

### Process optimization for the generation of furfural

**Fig. 5.**
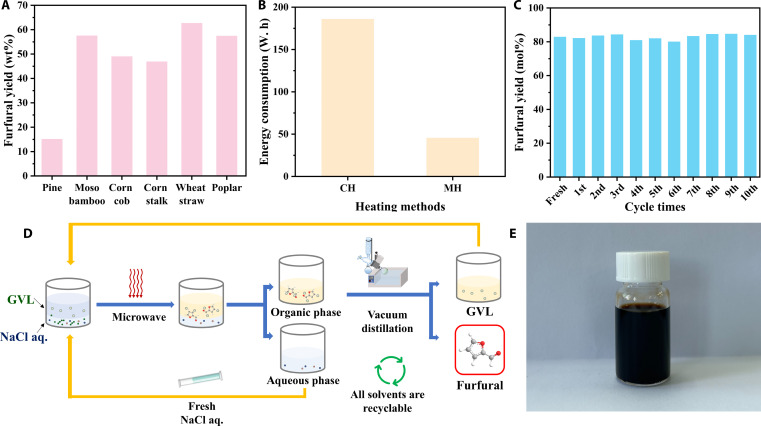
(A) Furfural yield from lignocellulosic biomass in the GVL/NaCl aq. biphasic system. (B) The comparison of energy consumption between microwave heating and conventional heating. (C) Reusability of the SnCl_4_ and GVL/NaCl aq. system. (D) Flowchart of the reusability of the SnCl_4_ and GVL/NaCl aq. system. (E) The physical diagram of furfural.

The conversion of real lignocellulosic biomass under optimal microwave-heating conditions was evaluated. As shown in Fig. 5A, wheat straw afforded the highest furfural yield of 62.72 wt% (on a hemicellulose basis), followed by moso bamboo at 57.59 wt% (the composition of the feedstock is shown in Table S9). Table S10 provides an analysis of the mass balance of liquid and solid components following the reaction. The results indicate that furfural is the predominant component produced from untreated biomass feedstock in this system and is efficiently extracted into the organic phase. As a result of the efficient conversion of pentoses, glucose emerged as the predominant component in the aqueous phase. Simultaneously, the analysis of the solid residue following the reaction revealed that it contained only glucose and lignin components. This finding demonstrates that the system can effectively convert lignocellulosic biomass into furfural.

Figure 5B compares the energy consumption of microwave versus oil-bath heating under optimal conditions for furfural production. Microwave irradiation resulted in the generation of 85.38 mol% furfural within 20 min, utilizing 0.046 kW·h of electricity. In contrast, oil-bath heating produced 80.06 mol% furfural over a period of 50 min, consuming 0.186 kW·h of electricity. This analysis reveals that microwave irradiation yields 0.68 mmol of furfural per kilojoule of electricity, while oil-bath heating yields only 0.16 mmol of furfural per kilojoule. Although raw-material procurement and labor remain the largest cost drivers, this result highlights microwave processing’s potential to cut operating expenses. Future techno-economic analyses will include transportation and storage costs.

Recoverability tests of the GVL/NaCl aq. system containing SnCl₄ showed excellent recyclability. In each cycle, the furfural is distilled off from the GVL layer and purified over activated carbon to remove humins; the aqueous phase is replenished with fresh solution and then reused. Over 10 consecutive cycles (Fig. 5C), furfural yields remained above 80 mol%, confirming sustained catalyst activity and solvent stability. Trace formic acid generated in situ buffers the aqueous phase, further promoting furfural formation [58]. The process flowchart for reuse and the physical diagram of furfural are shown in Fig. 5D and E, respectively.

Based on the properties of the reactants, we developed an innovative microwave-coupled reaction system that facilitates the mild and efficient conversion of xylan to furfural at the laboratory scale. This system establishes a valuable experimental foundation for the future continuous conversion of microwave-coupled reactants. In contrast to batch reactions, continuous reactors allow for a steady supply of fresh reactants, ongoing reaction processes, and prompt product separation. This methodology not only lowers costs but also improves product yields, making continuous production modes essential for practical industrial applications. The design challenges associated with continuous flow processes under microwave irradiation include achieving synchronous amplification of the electric field within the reactor, accurately controlling temperature gradients within the microwave field, selecting appropriate reactants, preventing pipeline blockages, and recovering catalysts. Addressing these challenges is essential for the continuous conversion of microwave-coupled hemicellulose. Future developments should focus on integrating computational chemistry with microwave technology, creating devices for precise temperature measurement, and reactant screening based on their microwave behavior, in order to predict and control chemical reactions within the microwave field. Such advancements will notably expedite the transition from laboratory to industrial applications, further enhancing the high-value utilization of biomass.

## Conclusion

In summary, based on the characteristics of the reactants, we designed experiments and screened systems from a microwave perspective. We proposed screening principles more suitable for microwave conditions and obtained certain patterns and conclusions. Microwave irradiation has been demonstrated to enhance the in situ extraction of furfural, increasing its partition coefficients from 22.7 to 29.3 under conventional heating conditions to 31.1 to 35.7. The role of microwave power in the depolymerization of xylan glycosidic bonds, as well as in the isomerization–dehydration process of xylose, is elucidated through the analysis of reaction kinetics. The application of high microwave power during the initial hydrolysis stage rapidly depolymerizes the glycosidic bonds in xylan, leading to the swift release of 87.9 mol% xylose. Following this, a subsequent low-power dehydration step facilitates furfural formation and its immediate transfer into the GVL phase, thereby minimizing side reactions and product loss. Under optimized conditions (140 °C, 20 min), this protocol achieves an 85.38 mol% furfural yield from xylan, exceeding the 78.1 mol% obtained in 120 min under conventional heating. The system maintains over 80 mol% furfural yield across 10 recycle cycles, and trials with real lignocellulosic biomass confirm low overall energy consumption due to the strong microwave absorbance of the GVL/NaCl aqueous medium. Overall, this approach offers a scalable, energy-efficient route to high-yield furfural production with integrated product separation and solvent–catalyst recyclability.

## Materials and Methods

### Materials

Xylan (from corn cob, 95%) was obtained from J&K Scientific Ltd. Corn cob, corn stalk, poplar, pine, moso bamboo, and wheat straw were collected from Jiangsu, Zhejiang, and Shandong Province, respectively, which were first chopped and milled, and then passed through a 120-mesh sieve, followed by drying at 105 °C for 24 h, reserved as the real lignocellulosic biomass substrates. Xylose and xylulose were obtained from Mackin Co. Metal salts, PC (99%), MIBK (≥99%), EA (≥99.5%), EtOH (99.5%), GVL (98%), and TL (≥99.5%) were purchased from Sinopharm Chemical Reagent Co., Ltd. (Shanghai China). All chemicals used in the experiments were of analytical purity and used without further purification.

### Production of furfural

A 35-ml sealed glass vessel was used to perform the reaction in a microwave reactor (CEM Discover 2.0). Experiments on the conversion of pentoses used SnCl_4_ as the Lewis acid catalyst in GVL/NaCl aq. biphasic systems. In a typical experiment, 0.2 g of xylan and 0.04 M SnCl_4_ were first placed in the container. After the addition of 6 ml of the organic solvent and 2 ml of 15 wt% NaCl aq., the reaction mixture was heated to the target temperature of 140 °C under the power 150 W and held for a while. The zero time was defined as the mixed reaction solution reaching the target temperature. After the reactions, the reactor was cooled to ambient temperature with the assistance of an air compressor by blowing air, and the mixture was filtered through a 0.22-μm membrane to filter the solid product. For all biphasic experiments, the organic and aqueous phases were separated and filtered with 0.22-μm filter membranes before analysis. The catalytic conversion of furfural in conventional heating was completed under oil-bath conditions. In a typical experiment, the same mixture as under microwave conditions was placed in a Teflon container with a reaction kettle, and then the reaction kettle was placed in an oil-bath. The other steps were consistent with those under microwave conditions.

### Product analysis

The obtained furfural in the organic phase was measured by gas chromatography (2010Plus, Shimadzu), equipped with a flame ionization detector and an SH-RTX-5 column (30 m × 0.32 mm × 0.5 μm). The gas chromatograph was used to determine the volatile components with 0.43 ml/min of gaseous helium as the carrier gas. The temperature of the injector was set to 260 °C, and the temperature of the detector was 280 °C. The initial temperature of the chromatographic column was set at 100 °C for 2 min, then heated up to 280 °C at the rate of 8.0 °C/min, and held at 280 °C for 10 min.

The furfural (FAL) yield was calculated using the following equations:

$$\begin{array}{c} \mathrm{FAL}\;\text{yield}\;\left(\text{from xylan, mol\%}\right)\\ =\frac{\text{moles of}\;\mathrm{FAL}\;\text{produced}}{\text{moles of pentoses from xylan}}\times 100\% \end{array}$$ (2)

$$\begin{array}{c} \mathrm{FAL}\;\text{yield}\;\left(\text{from lignocellulosic biomass, wt\%}\right)\\ =\frac{\text{weight of}\;\mathrm{FAL}\;\text{produced}}{\text{weight of starting xylan}\;}\times 100\% \end{array}$$ (3)

### Chemical composition and sugar analysis

The chemical composition of biomass raw materials was determined using standard biomass analysis methods provided by the National Renewable Energy Laboratory in the United States. Qualitative and quantitative analysis of soluble monosaccharides produced by acid hydrolysis of raw materials was performed using high-performance liquid chromatography (HPLC) (Waters E2695) equipped with a Biorad Aminex HPX-87H column (300 × 7.8 mm) and a refractive index detector (Waters 2414). The eluent was 5 mM diluted H_2_SO_4_ solution at a flow rate of 0.6 ml/min. The temperature of the detector and column oven was maintained at 40 and 55 °C, respectively. The content of soluble lignin was determined using an ultraviolet–visible spectrophotometer at a wavelength of 205 nm.

### ESI-MS

ESI-MS spectra were recorded using a ThermoScientific Q Exactive High-Resolution Mass Spectrometer. All samples were loop injected (2 μl), and ultrapure water was used as a mobile phase at a flow rate of 200 μl/min. The Q Exactive HESI source was operated in full MS in positive mode. The mass resolution was tuned to 140,000 full width at half maximum (FWHM) at *m*/*z* 200. The spray voltage was set to 3.50 kV, and the sheath gas and auxiliary gas flow rates were set to 45 and 15 arbitrary units, respectively. The transfer capillary temperature was held at 250 °C, and the S-Llens RF level was set at 50 V. Xcalibur software was used for data acquisition and processing.

## Data Availability

Data supporting the findings of this study are available in the main text or the Supplementary Materials.

## Supplementary Materials

Supplementary 1 Figs. S1 to S6 Tables S1 to S10 Scheme S1
