# Supplementary material for: Microwave-Derived Hierarchic Liquefaction of Pentose and Intensified Separation of Furfural
Source: Research (Wash D C). 2025 Nov 20;8:1008. doi: 10.34133/research.1008 (PMC12630080; doi:10.34133/research.1008)
Supplement: Supplementary 1 — Figs. S1 to S6 Tables S1 to S10 Scheme S1 [file research.1008.f1.docx]

Supplementary Information for

Microwave-Derived Hierarchic Liquefaction of Pentose and Intensified Separation of Furfural

Ruixuan Yao ^1^, Xiao Jiang ^1^, Jianchun Jiang ^1,2^, Kui Wang ^1,2,*^

1 Institute of Chemical Industry of Forest Products, Chinese Academy of Forestry; Key Lab. of Biomass Energy and Material, Jiangsu Province; International Innovation Center for Forest Chemicals and Materials; Jiangsu Co-Innovation Center of Efficient Processing and Utilization of Forest Resources, Nanjing, 210042, China

2 Institute of Biomass Science and Engineering, Henan University of Technology, Zhengzhou, 450000, China

*Address correspondence to: wangkui@caf.ac.cn

**1. Supplementary figures**

**
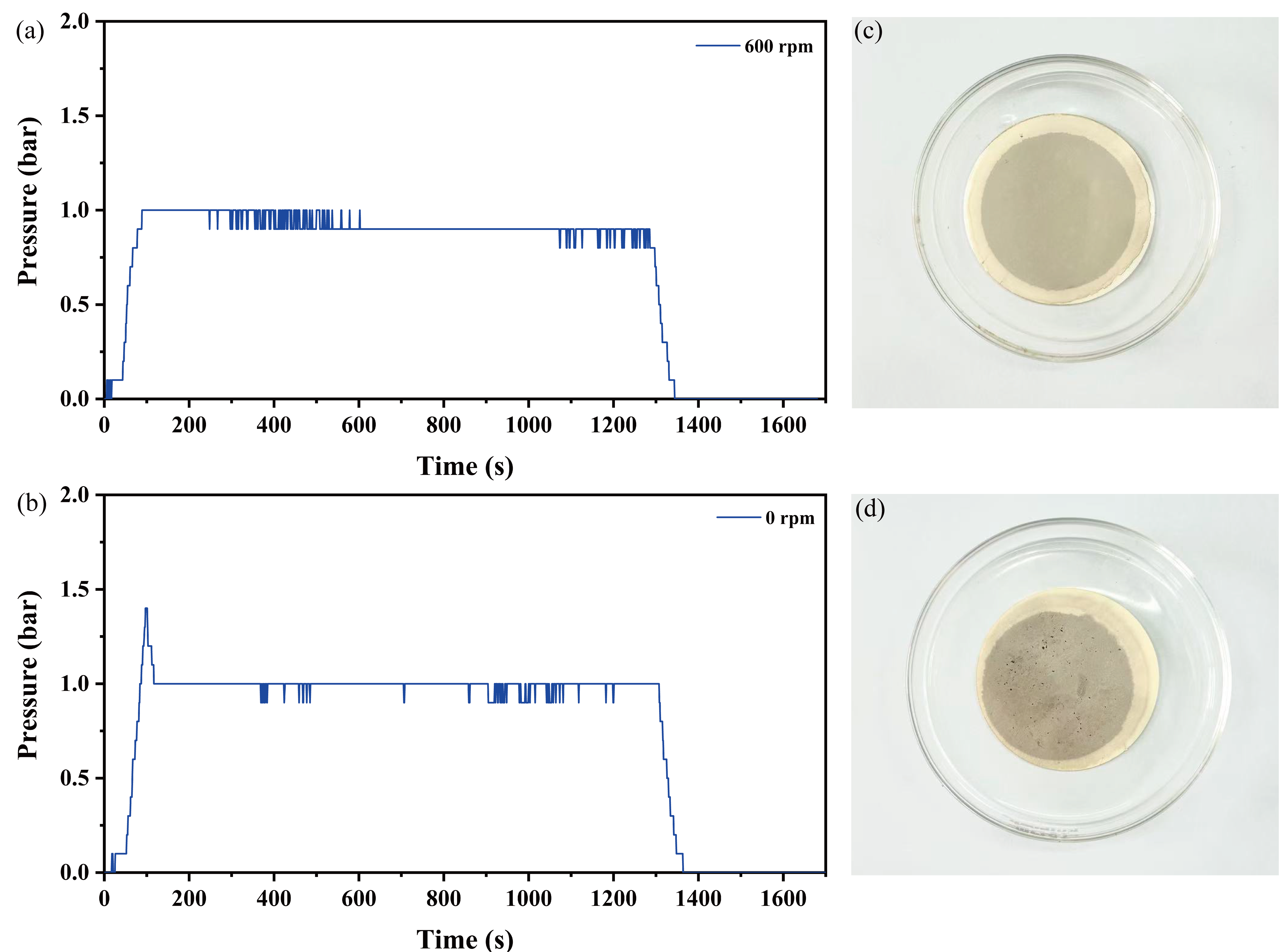
**

Fig. S1 Reaction pressure at (a) 600 rpm and (b) 0 rpm. Humins in the reaction at (c) 600 rpm and (d) 0 rpm.

**
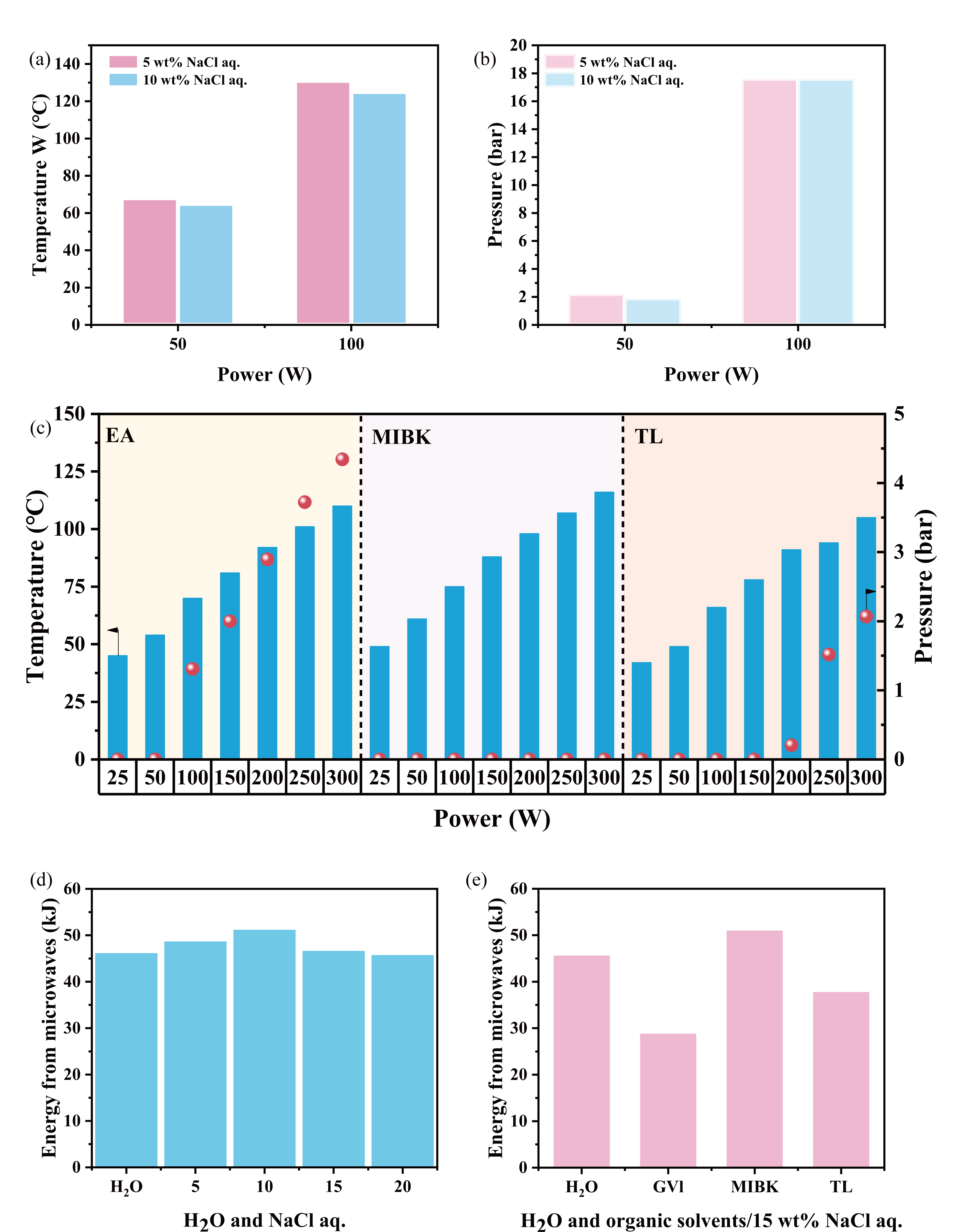
**

Fig. S2 The power consumed by different concentrations NaCl aq. to reach (a) temperatures and (b) pressure. (c) The power consumed by organic solvents to reach temperatures and pressure. Energy consumption of (d) different concentrations NaCl aq. and (e) organic solvents/15 wt% NaCl aq.


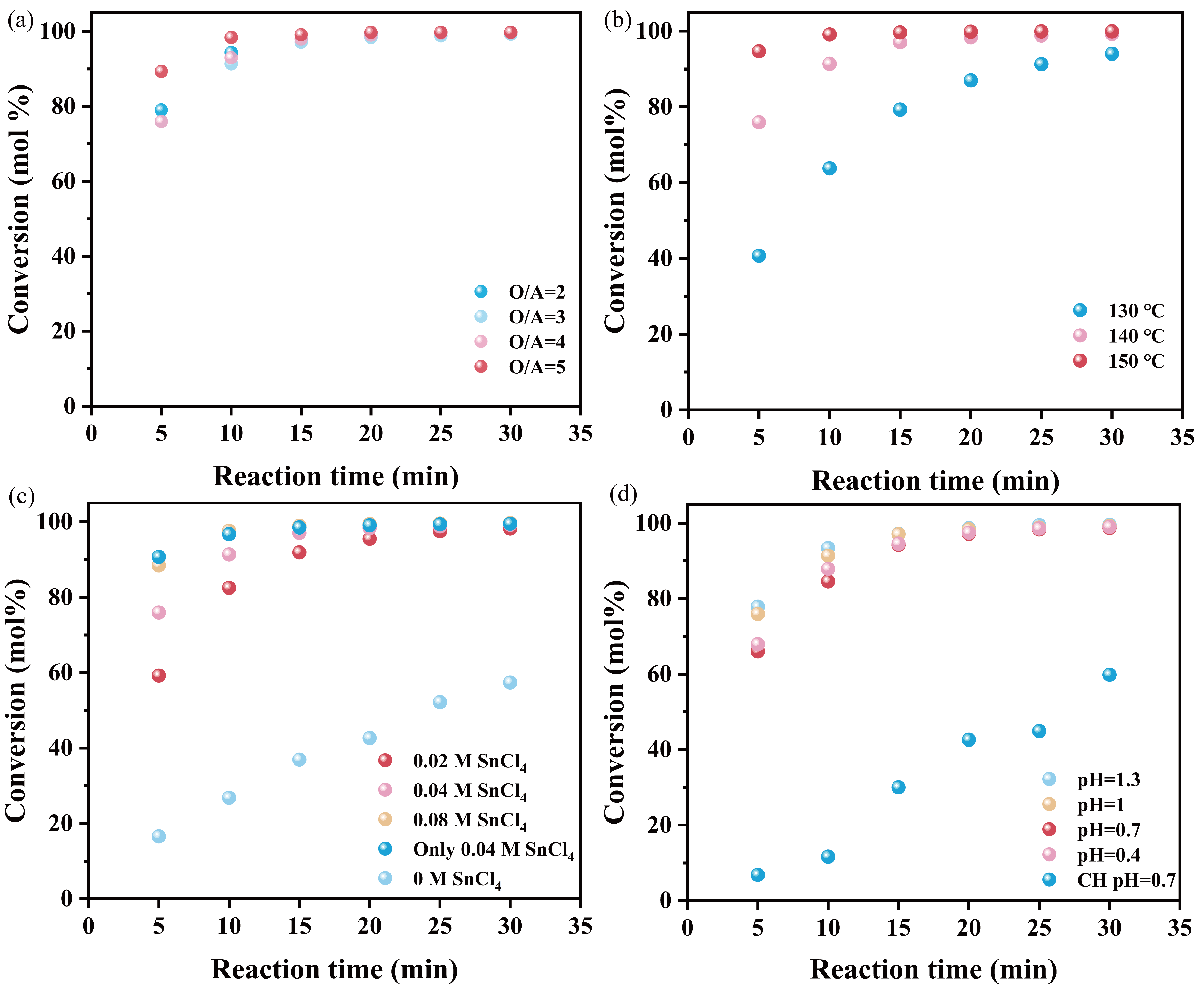


Fig. S3 The effects of the conversion of xylan with (a) different solvent ratios, (b) temperature, (c) Lewis acid concentrations, and (d) pH value.


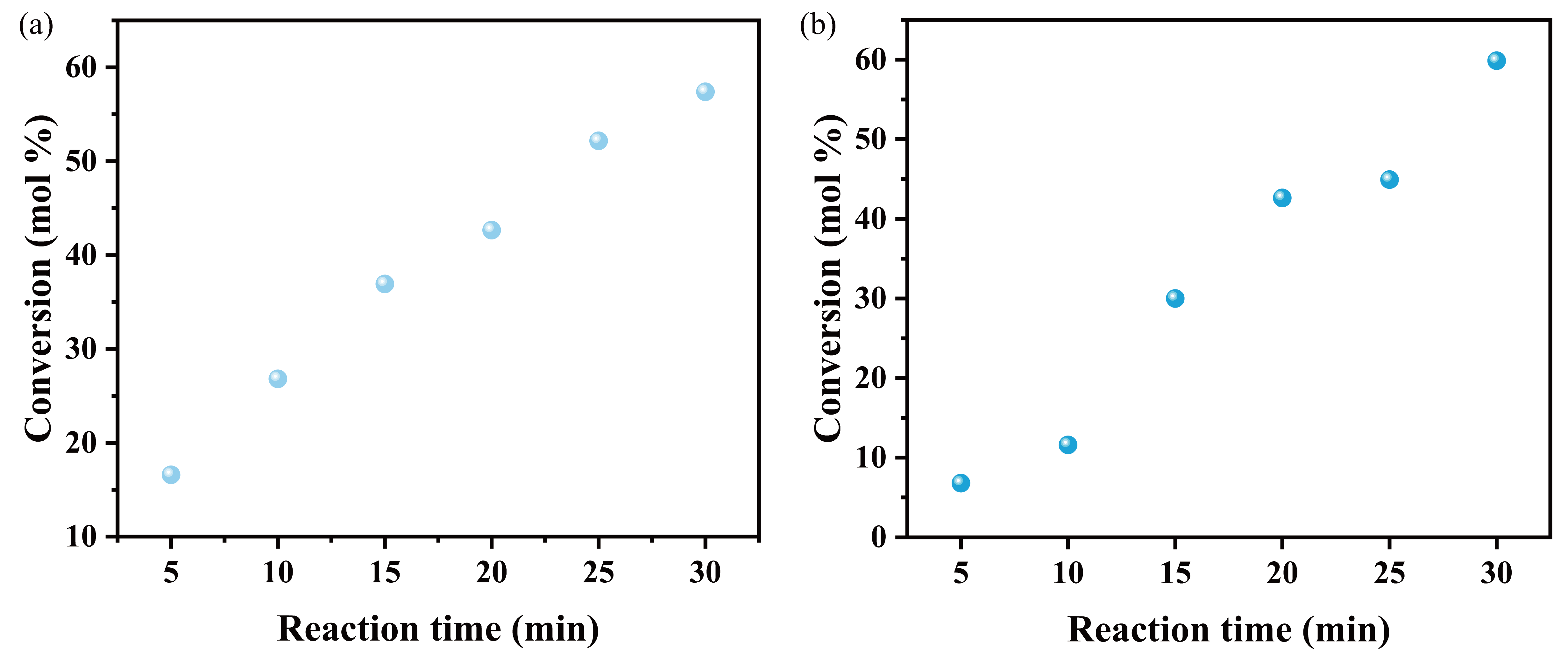


Fig. S4 The conversions of xylan to furfural (a) without SnCl_4_ and (b) under conventional heating.


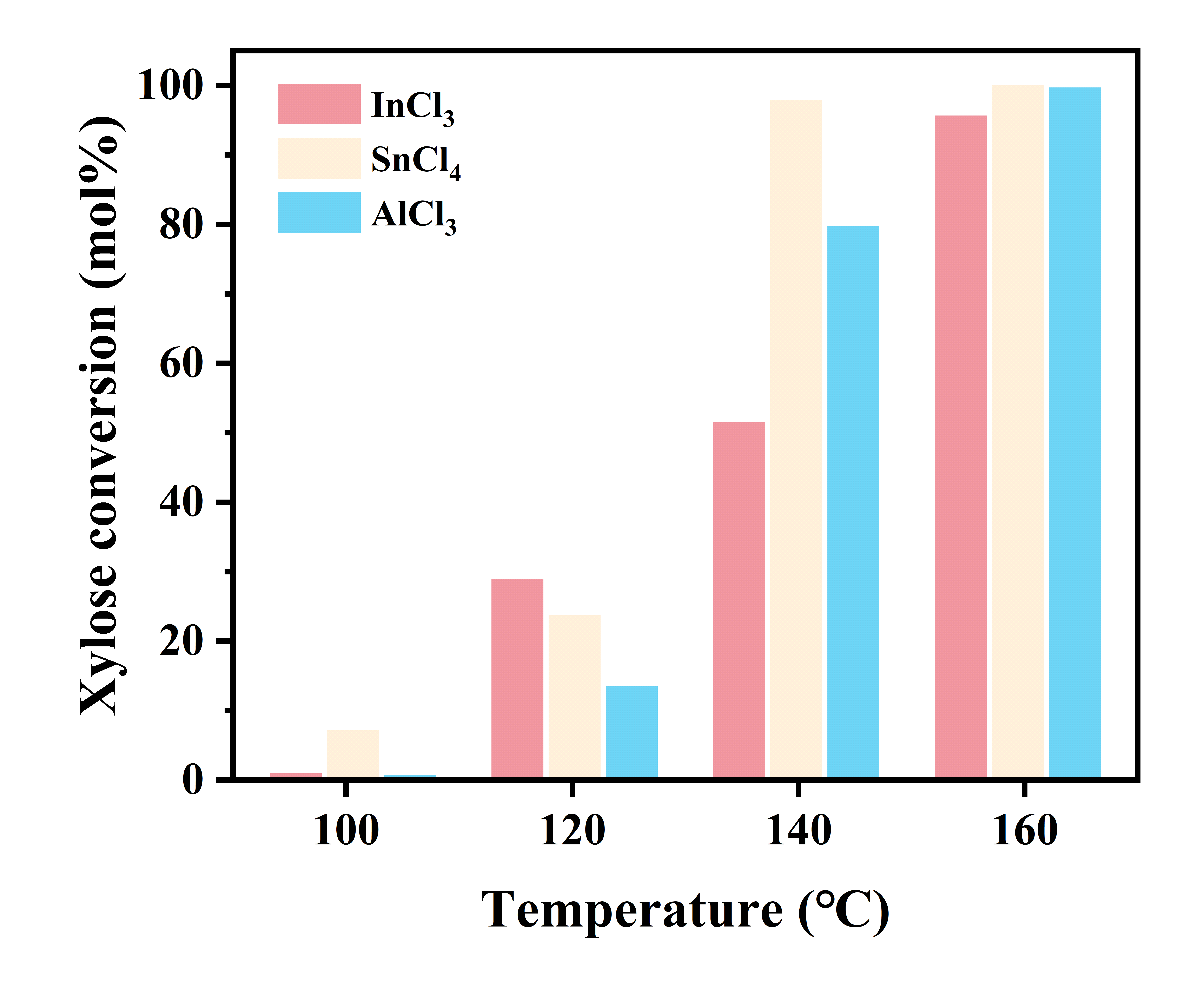


Fig. S5 Conversion rates of xylose to furfural at different temperatures.


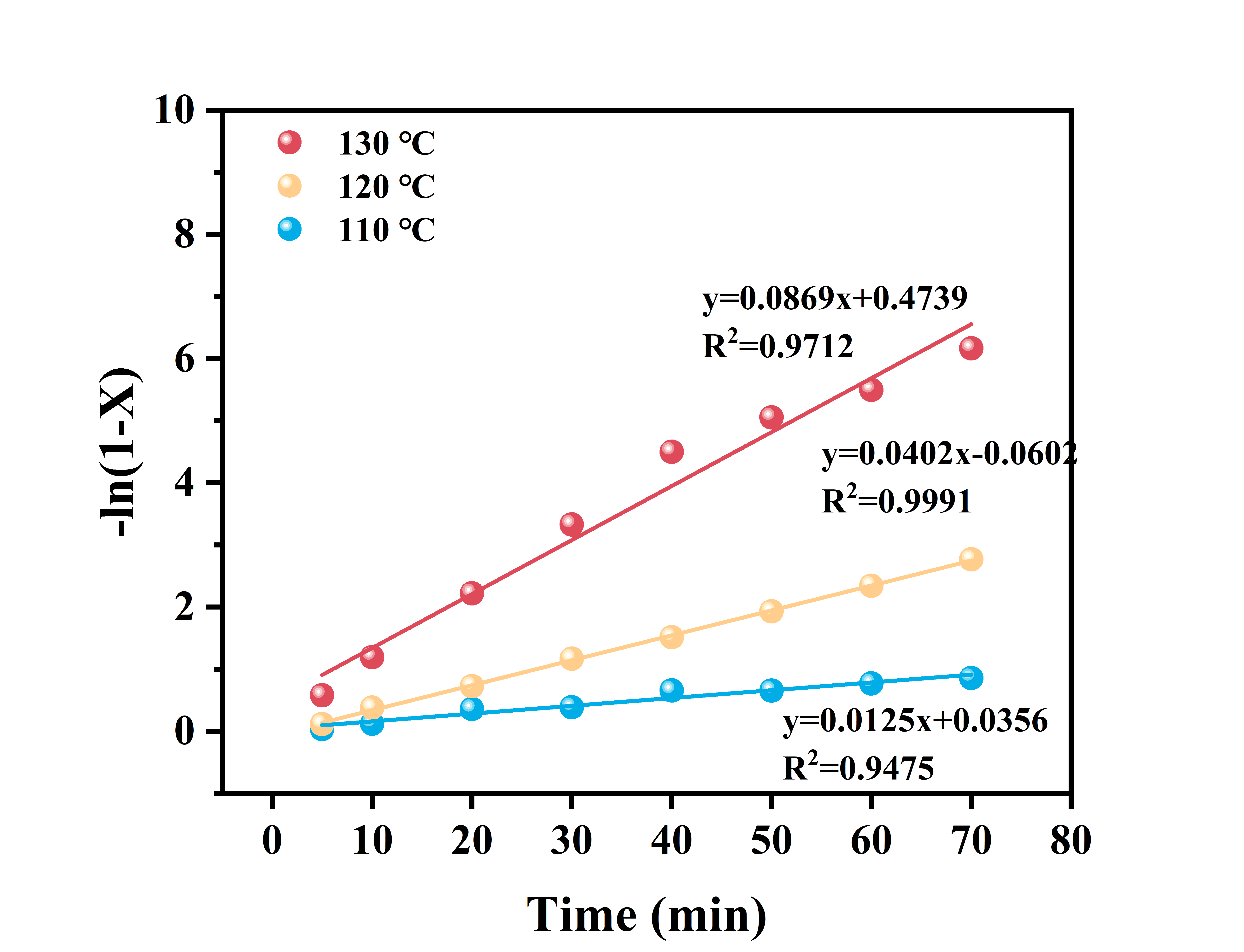


Fig. S6 The rate constant for the conversion of xylose to furfural.

**2. Supplementary Table**

Table S1 The dielectric properties of solvents

| Solvents | ε′ | ε″ | tanδ |
| --- | --- | --- | --- |
| γ- valerolactone | 24.71 | 13.84 | 0.56 |
| 15 wt% NaCl aq. | 46.54 | 50.26 | 1.08 |
| H_2_O | 70.52 | 23.27 | 0.33 |

ε′: dielectric constant

ε″: dielectric loss

tan*σ*: the tangent value of the dielectric loss angle

Table S2 The conversion of xylan to furfural at a holding time of 0 min when reaching the target temperature of 130℃

| Substrate | Catalysts | | Xylose yield (mol%) | | Xylulose yield (mol%) | | Furfural yield (mol%) |
| --- | --- | --- | --- | --- | --- | --- | --- |
| xylan | SnCl_4_ | 26.85 | | 0 | | 16.56 | |
|  | AlCl_3_ | 30.07 | | 0 | | 9.62 | |
|  | InCl_3_ | 36.29 | | 0 | | 7.91 | |

Table S3 The conversion of xylose to furfural at a holding time of 0 min when reaching the target temperature

| Substrate | Temperature (℃) | Catalysts | Conversion rate (mol%) | Furfural yield (mol%) |
| --- | --- | --- | --- | --- |
| Xylose | 140 | SnCl_4_ | 69.37 | 52.91 |
|  |  | AlCl_3_ | 51.91 | 22.14 |
|  |  | InCl_3_ | 37.71 | 13.13 |

Table S4 The conversion of xylulose to furfural at a holding time of 0 min when reaching the target temperature

| Substrate | Temperature (℃) | Catalysts | Conversion rate (mol%) | Furfural yield (mol%) |
| --- | --- | --- | --- | --- |
| Xylulose | 140 | SnCl_4_ | 100 | 74.76 |
|  |  | AlCl_3_ | 100 | 72.58 |
|  |  | InCl_3_ | 100 | 72.49 |

Table S5 The heating time for the hydrolysis of xylan at different powers

| Power (W) | 45 | 50 | 100 | 150 | 200 | 250 |
| --- | --- | --- | --- | --- | --- | --- |
| Time (s) | 317 | 256 | 136 | 91 | 79 | 72 |

**
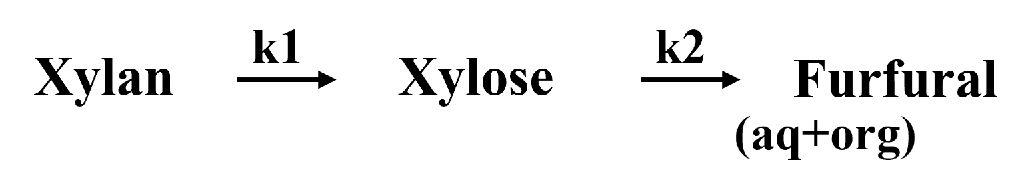
**

Scheme. S1 Reaction kinetic modeling of xylan conversion to furfural under microwave heating conditions.

Kinetic study

The kinetic studies were investigated by varying the reaction temperature (383-403 K) and recording the yields of different products within 5 min of reaction time. Since the reaction time was short, the degradation of xylan in this time was neglected, and the conversion of xylan was calculated based on the yields of xylose, xylulose, and furfural. According to the overall reaction model (see Scheme 1 and Scheme S1), the reaction rate equations of xylan conversion in H_2_O are described as eqs (1) - (2):

| $\frac{\boldsymbol{d}\boldsymbol{C}_{\boldsymbol{xylan}}}{\boldsymbol{dt}}\boldsymbol{=-k}\boldsymbol{1}\boldsymbol{C}_{\boldsymbol{xylan}}$ | (1) |
| --- | --- |

| $\frac{\boldsymbol{d}\boldsymbol{C}_{\boldsymbol{xylose}}}{\boldsymbol{dt}}\boldsymbol{=k}\boldsymbol{1}\boldsymbol{C}_{\boldsymbol{xylan}}\boldsymbol{-k}\boldsymbol{2}\boldsymbol{C}_{\boldsymbol{xylose}}$ | (2) |
| --- | --- |

Table S6 The rate constant for the hydrolysis of xylan to furfural

| Substrate | Temperature (℃) | k1 | k2 |
| --- | --- | --- | --- |
| xylan | 110 | 0.709903293 | 0.005930638 |
|  | 120 | 0.642651631 | 0.021121183 |
|  | 130 | 40.193534368 | 0.044096903 |

Table S7 The rate constant for the conversion of xylose to furfural

| Substrate | Temperature (℃) | k |
| --- | --- | --- |
| xylose | 110 | 0.0125 |
|  | 120 | 0.0402 |
|  | 130 | 0.0869 |

Table S8 Comparison with the results of the furfural production process reported in the literature

| Entry | Substrate | Catalysts | Solvents | Heating  method | Temperature (℃) | Time  (min) | | Furfural yield  (%) | Ref |
| --- | --- | --- | --- | --- | --- | --- | --- | --- | --- |
| 1 | xylan | SnCl_4_ | GVL/NaCl aq. | Microwave | 140 | 20 | 85.38 | | This work |
| 2 | xylose | IL | H_2_O/GVL | Convention | 140 | 120 | 79.76 | | [8] |
| 3 | arabinose | IL | H_2_O/GVL | Convention | 140 | 120 | 58.7 | | [8] |
| 4 | xylose | EC-SGO_2_M | H_2_O | Convention | 180 | 60 | 76.62 | | [48] |
| 5 | corncob | (SO_4_)^2-^/SnO^2-^diatomite | H_2_O/GVL | Convention | 170 | 30 | 68.9 | | [49] |
| 6 | xylose | (SO_4_)^2-^/CX-DMSn | H_2_O | Convention | 180 | 300 | 66.24 | | [50] |
| 7 | corncob | (SO_4_)^2-^/CX-DMSn | H_2_O | Convention | 180 | 300 | 65.74 | | [50] |
| 8 | eucalyptus | AlCl_3_ | DES/MIBK | Convention | 140 | 90 | 70.3 | | [51] |
| 9 | corncob | (SO_4_)^2-^/SiO^2-^Al_2_O_3_/La^3+^ | H_2_O | Convention | 190 | 60 | 21 | | [52] |
| 10 | xylose | CO_2_ | H_2_O/THF/MIBK | Convention | 180 | 60 | 56.6 | | [53] |
| 11 | xylose | maleic acid | H_2_O | Microwave | 200 | 28 | 67 | | [54] |
| 12 | xylan | HCl | IL | Microwave | 140 | 1 | 78.8 | | [55] |
| 13 | xylose | HCl | H_2_O | Microwave | 170 | 30 | 40 | | [56] |
| 14 | xylose | HCl | H_2_O/MIBK | Microwave | 160 | 30 | 50 | | [56] |
| 15 | xylose | / | H_2_O | Microwave | 200 | 60 | 49 | | [57] |
| 16 | xylan | / | H_2_O | Microwave | 180 | 120 | 17.5 | | [57] |

Given that the formation of furfural in this study is significantly influenced by temperature and time, the quantity of furfural produced under the optimal temperature and time conditions reported in the literature was calculated for comparative analysis using the following equations:

| $\boldsymbol{R}_{\boldsymbol{0}}\boldsymbol{=t\times}\boldsymbol{e}^{\frac{\boldsymbol{T-}\boldsymbol{T}_{\boldsymbol{ref}}}{\boldsymbol{Q}_{\boldsymbol{10}}}}$ | (3) |
| --- | --- |
| $\boldsymbol{Q}_{\boldsymbol{10}}\boldsymbol{=}\frac{\boldsymbol{k(T+10)}}{\boldsymbol{k(T)}}$ | (4) |
| $\boldsymbol{k=}\frac{\boldsymbol{Yield}}{\boldsymbol{t}}$ | (5) |

Where t, T and T_ref_ represent the reaction time (min), temperature, and room temperature (℃), respectively.

Table S9 Accounts of the three major elements in 300 mg lignocellulosic biomass

| Sample | Cellulose (mg) | Hemicellulose (mg) | Lignin (mg) |
| --- | --- | --- | --- |
| Corn cob | 103.7 | 74.94 | 46.61 |
| Pine | 112.91 | 55.60 | 82.65 |
| Moso bamboo | 108.60 | 50.21 | 85.97 |
| Corn stalk | 77.57 | 46.93 | 59.49 |
| Wheat straw | 67.95 | 35.33 | 61.75 |
| Poplar | 126.84 | 35.33 | 76.09 |

Table S10 Product composition from direct conversion of 200 mg untreated biomass feedstock

| Substrate | Organic phase (mg) | | |  | Aqueous phase (mg) | |  | Residues (mg) | | | | |
| --- | --- | --- | --- | --- | --- | --- | --- | --- | --- | --- | --- | --- |
|  | Furfural | 5- Hydroxymethyl furfural | Levulinic acid |  | Glucose | Xylose |  | Total | Glucose | Xylose | Lignin | Others |
| Corn cob | 24.50 | 6.20 | 2.35 |  | 15.42 | 3.83 |  | 44.40 | 26.86 | 0 | 5.49 | 12.05 |
| Pine | 5.63 | 3.11 | 5.13 |  | 13.23 | 8.14 |  | 65.30 | 36.61 | 0 | 17.92 | 10.77 |
| Moso bamboo | 19.28 | 5.71 | 2.12 |  | 16.22 | 1.80 |  | 51.15 | 35.98 | 0 | 13.78 | 1.39 |
| Corn stalk | 14.70 | 5.13 | 1.84 |  | 9.04 | 3.51 |  | 52.32 | 31.15 | 0 | 11.79 | 9.41 |
| Wheat straw | 16.20 | 2.66 | 5.91 |  | 9.93 | 2.99 |  | 58.05 | 33.12 | 0 | 13.64 | 11.29 |
| Poplar | 13.54 | 1.66 | 5.45 |  | 16.47 | 2.45 |  | 68.25 | 38.24 | 0 | 16.26 | 13.75 |
